# Fetal response to placental dysfunction leads to 5-hmeC and H2A.Z/H3 acetylation in NOS3 while short-termed mRNA boost entails deferred self-attenuation

**DOI:** 10.1186/2194-7791-1-S1-A2

**Published:** 2014-09-11

**Authors:** Andreas Jenke, M Kanders, S Forcob, R Willems, S Wirth, J Postberg

**Affiliations:** HELIOS Children´s Hospital, Witten/Herdecke University, Witten, Germany

## Background

Substantial epidemiological evidence showed that an adverse intrauterine environment leads to permanent changes in physiology subsequently influencing the risk for adult diseases.

## Results

In human fetuses NOS3 expression directly correlated with 5-hydroxymethylcytosine levels as well as H3K9ac and H2A.Zac at the transcription start site. Using an in vitro model for placental dysfunction we confirmed the dynamic NOS3 response and histone acetylation patterns. Concomitantly we recognized massive turnover of Stat3 at a discrete binding site in the NOS3 promoter upon hypoxic conditions. Moreover, knock-down experiments targeting either STAT3 or NOS3 provided strong evidence for a functional NOS3 and STAT3 relationship. Interestingly, induced hyperacetylation and in vitro reporter assays showed that NOS3 expression becomes self-attenuated by co-expression of intronic 27-nt-ncRNA modulating STAT3 signaling.

## Conclusion

We demonstrate that adaptive NOS3 regulation in response to placental dysfunction involves differential epigenomic signatures and that a rapid NOS3 self-limiting response to an ectopic trigger co-exists with longer-termed epigenetic changes. Persistence of such marks might contribute to impaired vascular endothelial response and increased risk for diseases later in life of children who suffered from placental insufficiency.

